# The long-range correlation and evolution law of centennial-scale temperatures in Northeast China

**DOI:** 10.1371/journal.pone.0198238

**Published:** 2018-06-06

**Authors:** Xiaohui Zheng, Yi Lian, Qiguang Wang

**Affiliations:** 1 College of Climate Change and Earth System Science, Beijing Normal University, Beijing, China; 2 Institute of Jilin Meteorological Science, Changchun, China; 3 China Meteorological Administration Training Center, CMA, Beijing, China; Universidade de Aveiro, PORTUGAL

## Abstract

This paper applies the detrended fluctuation analysis (DFA) method to investigate the long-range correlation of monthly mean temperatures from three typical measurement stations at Harbin, Changchun, and Shenyang in Northeast China from 1909 to 2014. The results reveal the memory characteristics of the climate system in this region. By comparing the temperatures from different time periods and investigating the variations of its scaling exponents at the three stations during these different time periods, we found that the monthly mean temperature has long-range correlation, which indicates that the temperature in Northeast China has long-term memory and good predictability. The monthly time series of temperatures over the past 106 years also shows good long-range correlation characteristics. These characteristics are also obviously observed in the annual mean temperature time series. Finally, we separated the centennial-length temperature time series into two time periods. These results reveal that the long-range correlations at the Harbin station over these two time periods have large variations, whereas no obvious variations are observed at the other two stations. This indicates that warming affects the regional climate system’s predictability differently at different time periods. The research results can provide a quantitative reference point for regional climate predictability assessment and future climate model evaluation.

## Introduction

The Northeast region of China is an important industrial base and is the largest commercial grain producing area. Its vast territory is surrounded by mountain ranges on three sides and consists mainly of plains in the middle. It has very large climate variations and is one of the regions with notable global warming responses[1–3]. Over the past hundred years, climate change due to global warming has significantly impacted regional precipitation, temperature, and other meteorological variables. Cold events affect crop production significantly, especially during the growing season, e.g., frequent cold events in Northeast China from the 1950s to 1970s caused crop production to decrease up to 20% in many of those years and dramatically affected the safety of the food supply in China. At the same time, climate warming since the 20^th^ century has accelerated and has made regional climate prediction more difficult and disaster prevention and mitigation more challenging. Therefore, it is necessary to study the changing atmospheric system predictability in this region from a new angle and to target improvements in prediction accuracy.

The chaotic characteristics of the atmospheric system limit the prediction capabilities of numerical integration models, and the corresponding predictabilities to initial data may vary at different regions. When the main factors affecting the system status change, the temporal limitations of predictability can be very different[4–9]. Therefore, it is important to study the inherent law of system predictability to improve prediction efficiency realistically[10–14]. The atmospheric system is a very complex, dissipative, and non-diabatic nonlinear dynamic system and outputs an active time series of atmospheric variables. The mean values, variances, and other characteristic parameters from these time series vary with time so that the traditional methodology of analyzing the correlated characteristics of various time series has limitations. The detrended fluctuation analysis (DFA) method[15] is a newly developed scaling analysis tool that can effectively filter the trend components at each scale, treat non-stationary data, remove spurious correlation phenomenon, and test long-range correlations in a non-stationary time series[16–20]. The long-range correlation is also called the long memory property, or long-range persistence, and its characteristics include the self-correlation functions of a time series with a slow power law decay. This means that there are still significant correlations between long-separated time periods and that the past status can continue to affect the current and future statuses of the system. The DFA method has been successfully applied in many fields including noise analysis, medical physiology, geology, economics[21–24] and meteorology[25–30]. Generally speaking, the observation stations at Harbin, Changchun, and Shenyang are located in areas that represent the typical climate in the Northeast region. Therefore, this paper first analyzes the centennial-scale monthly mean temperature trends at the Harbin, Changchun, and Shenyang stations in the Northeast region. Then, based on these analyses, we study the long-range temperature correlation and evolution law and investigate the memory variations of the temperature time series in different months. The results provide a scientific basis for long-term temperature predictability research in the Northeast region.

## Data and methods

This paper utilized the monthly mean temperature time series from January 1909 to December 2014 at the Harbin, Changchun, and Shenyang stations in the Northeast region. These data were normalization test and correction by RHtest and t-test[15].

The DFA method is a tool used to study the existence of long-range correlations in a nonlinear time series and is widely applied to a variety of natural science and social science fields. Its computational procedure is as shown in the following steps[15,31]:

First, one needs to calculate the cumulative difference of the time series {*x*_*i*_, *i* = 1,2,⋯, *N*}:

$$Y(i)=\sum_{i=1}^{N}\left(x_{i}-\bar{x}\right)$$ (1)

where $\bar{x}=\frac{1}{N}\sum_{i=1}^{N}x_{i}$.

Second, *Y*(*i*) is separated into *N*_*s*_ number of intervals *v* with the same length *s* and *N*_*s*_ = [*N*/*s*].

Third, in every interval *v*, the data are fitted using the least squares method to calculate the local trend. The time series after the local trend is fitted is *Y*_*s*_(*i*), which represents the difference between the original time series and the fitted data:

$$Y_{s}(i)=Y(i)-P_{v}(i)$$ (2)

where *P*_*v*_(*i*) is the fitting polynomial in interval *v*.

Fourth, the variance after the trend fitting in every interval is calculated as

$$\begin{array}{cc} F^{2}(v,s)=\frac{1}{s}\sum_{i=1}^{s}Y_{s}^{2}[(v-1)s+i] & \left(v=1,2,\cdots ,N_{s}\right) \end{array}$$ (3)

Fifth, the standard DFA fluctuation is calculated by the square root of the mean variance in all the intervals with the same length:

$$F(s)=\sqrt{\frac{1}{N_{s}}\sum_{v=1}^{N_{s}}F^{2}(v,s)}$$ (4)

If the time series is long-range correlated, the DFA fluctuation *F*(*s*) has a power law relation with the lag time *s*

$$F(s)\propto s^{\alpha }$$ (5)

where in a double-logarithmic coordinate of (*F*(*s*),*s*) and by using the least square fitting, the slope of a straight line is the scaling exponent *α*. When 0 < *α* < 0.5, the time series is not correlated and only has short-term memory so that a current event has no influence on long-term future events; when *α* = 0.5, the original time series is white noise, such as Brownian motion; when 0.5 < *α* < 1.0, the time series has long-range correlation characteristics and long-term memory so that current and future events are correlated. Additionally, the bigger the scaling exponent *α* value is, the stronger the predictability of the time series.

## Analyses of the centennial-scale temperature variations in the Northeast region

**Fig 1:**
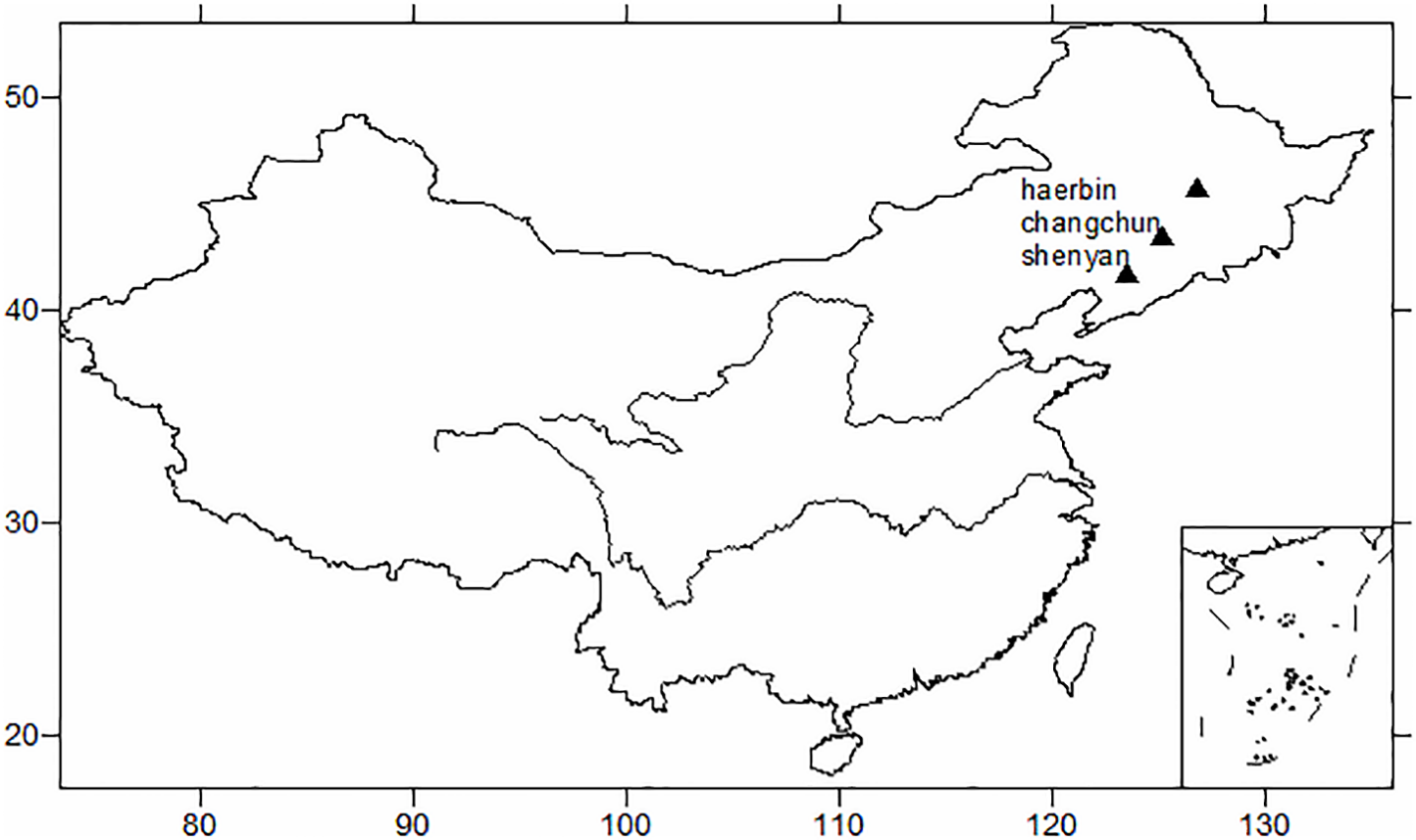
The location of the three stations.

In this section, the annual mean temperature is calculated from the monthly mean temperature time series from January 1909 to December 2014 at the three stations in Harbin, Changchun, and Shenyang(Fig 1). Then, the temperature variations at an interdecadal scale are compared and analyzed.

### Variations of centennial annual mean temperature in the Northeast region

**Fig 2:**
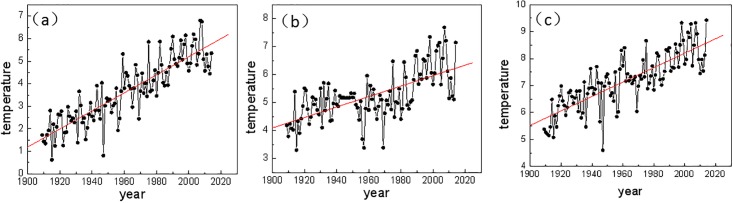
Annual mean temperature(units:°C) time series from 1909–2014 at (a) Harbin; (b) Changchun; (c) Shenyang.

Fig 2 shows the annual mean temperatures at Harbin, Changchun, and Shenyang along with their continuous increasing trends over the past 106 years, which is consistent with the background of global warming. The temperature shows the most obvious warming (Fig 2(a)) at Harbin and the least at Changchun (Fig 2(b)). Just after the 1960s, the temperature at Changchun started to show an obvious warming trend.

To compare the rates of the increasing temperatures at the three stations, the warming rates during the past 106 years. The warming rate is 0.040°C/a at the Harbin station, 0.019°C/a at the Changchun station, and 0.027°C/a at the Shenyang station. In addition, the figure and table show that the relative cold years have reduced and the relative warm years have increased. Two questions need to be answered: 1) does the monthly mean temperature also follow the same trend? 2) can cold events that affect the growing season still occur under global warming? In the next section, simple analyses are performed considering the monthly mean temperature variations.

### Variation characteristics of the centennial-scale monthly mean temperature in the Northeast region

In this section, the warming rates of the monthly mean temperatures during 106 years (from 1909–2014) at the Harbin, Changchun, and Shenyang stations are calculated. As shown in Fig 2, warming occurs during every month at the Harbin station (Fig 2(a)) with obvious warming during the winter and spring seasons. The warming rate at the Harbin station is the largest in March (0.059°C/a) and the smallest in August (0.017°C/a). At the Changchun station, (Fig 2(b)), the monthly mean temperature increases for most of the months, especially during the winter. The warming rate at Changchun is the largest in January (0.035°C/a) and the smallest in July (-0.003°C/a), with a weak cooling trend. Fig 2(c) shows the monthly temperature variations and the warming trend in all the months during the past 106 years at the Shenyang station, where the largest warming rate was in February (0.044°C/a) and the smallest in July (0.005°C/a).

From the above comparative analyses, the global warming trend during the past hundred years is quite evident in the Northeast region, especially with the strong signal in the winter season. Over the past 106 years, the mean temperature in March at Harbin has increased 6.5°C but with slower warming during the summer. The temperatures show less obvious warming in all three stations, especially in July. Therefore, one can conclude that the cold events during the growing season in the region are not significantly reduced. In the next section, we will study whether there are any obvious changes in the stability of the climate system in the Northeast region and any uncertain increase in the temperature prediction from the long-range correlation calculation.

## The long-range correlation and evolution of the centennial-scale temperatures in the Northeast region

The long-range correlation of the monthly mean temperatures at the three stations in the Northeast region is calculated first in this section. The scaling exponent for the monthly temperature time series is calculated and compared for a 106 year period. Then, the scaling exponent for the annual mean temperature is calculated, and its values at different time periods during the 106 years are calculated and compared.

### The long-range correlation of the monthly mean temperatures in the Northeast region

**Fig 3:**
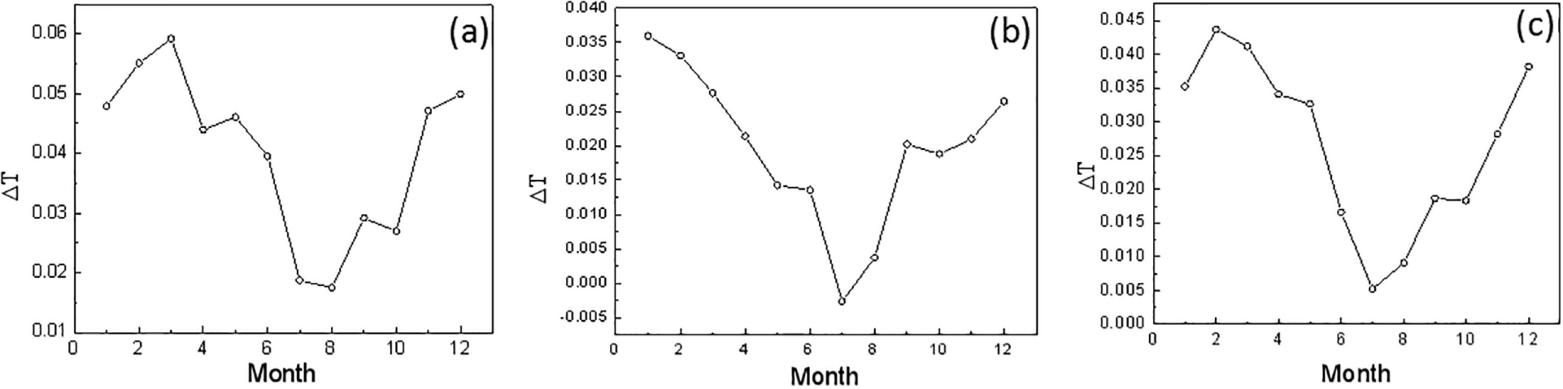
Warming rate of the monthly mean temperatures (units:°C/100a) from 1909–2014 at (a) Harbin; (b) Changchun; (c) Shenyang.

Fig 3 shows the distribution of the DFA fluctuation scaling rate for the monthly mean temperature data from the three stations in the Northeast region. The centennial temperature time series at the Harbin, Changchun and Shenyang stations all agree with the scaling rates. Although the previous statistical results stated obvious warming trends of the temperature time series and different annual variations from the three stations, Fig 3 represents the stable dynamic structural features of the atmospheric system in the Northeast region.

We calculated the scaling exponents of the monthly mean temperatures from 1909–2014 at the stations. The scaling exponent *α* is 0.74, 0.83, and 0.88 at Harbin, Changchun, and Shenyang, respectively, which are all larger than 0.5 and less than 1. This indicates that there is very good long-range correlation and predictability in the centennial-scale temperature time series at the three stations. The scaling exponent at the Shenyang station is the highest and is the lowest at the Harbin station. This may not relate to the warming rate at the centennial scale that was analyzed in the previous section. The long-range correlation of the atmospheric system is a rather independent feature that represents the stability status of the regional atmosphere to a certain degree.

**Fig 4:**
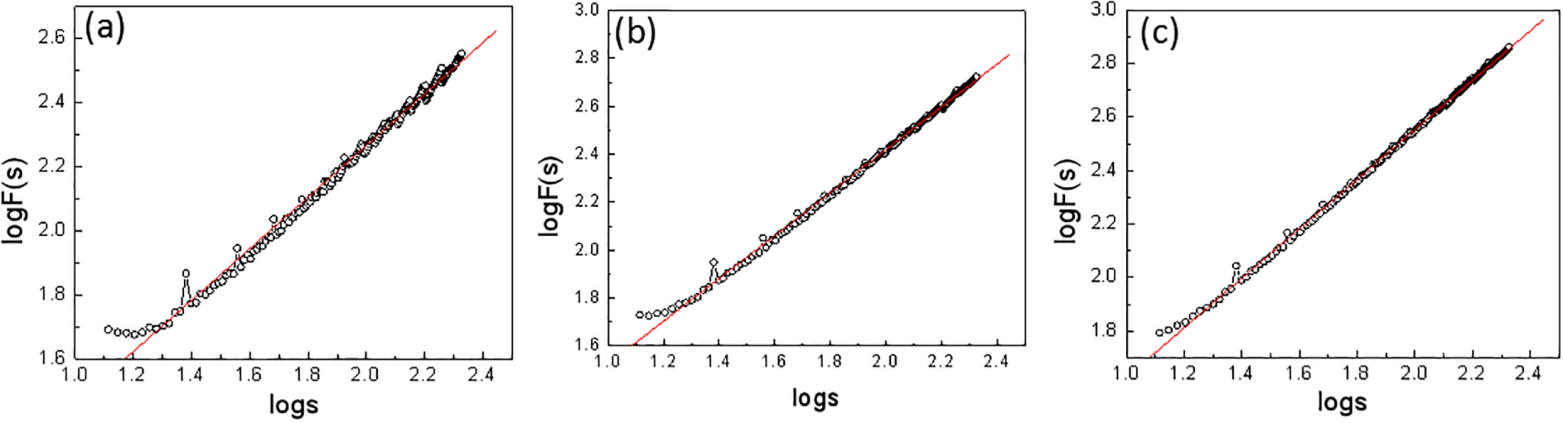
The scaling rate of the monthly mean temperatures during 1909–2014 at the three stations: (a) Harbin; (b) Changchun; (c) Shenyang.

**Fig 5:**
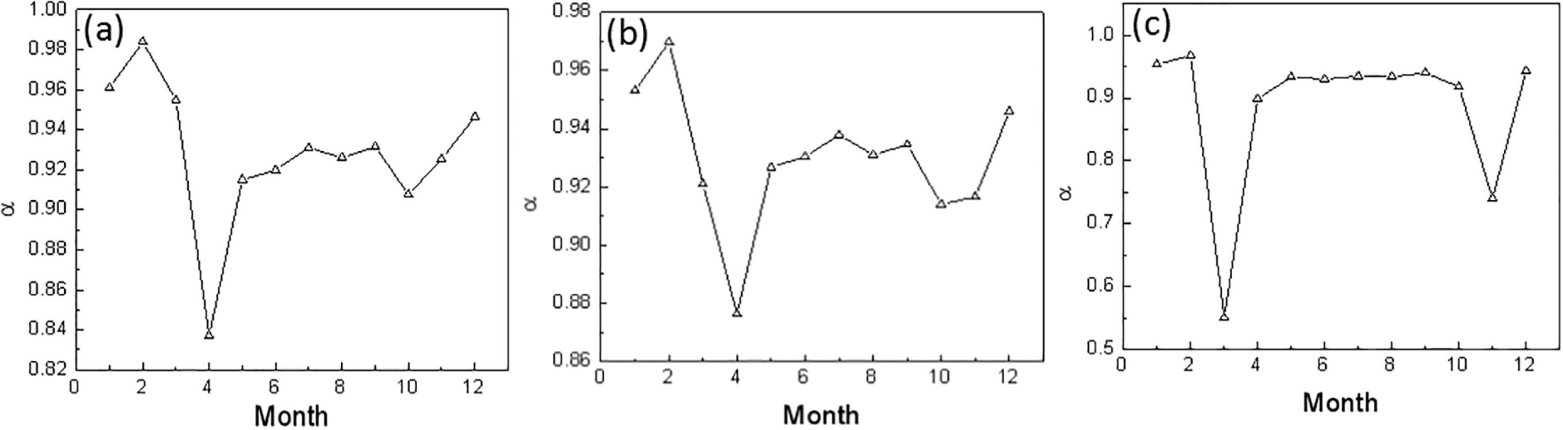
The scaling exponent of the monthly mean temperature from 1909–2014 at the three stations: (a) Harbin; (b) Changchun; (c) Shenyang.

The monthly scaling exponents for 1909–2014 at the three stations are calculated in order to study the evolution of the regional long-range correlation (Fig 4). The scaling exponents for the monthly mean temperature time series are different, as shown in the figure. Overall, the scaling exponents presented in April at the Harbin and Changchun stations are rather small, and the March scaling exponent (0.55) at Shenyang is also rather small. So, for the temperature time series, the instability of the temperature in the Northeast region is high, and its predictability in the spring is weak. This is consistent with the regional climate characteristics. For the other months, the scaling exponents at the three stations are all over 0.8 so that the predictability of the temperature time series during these months is much better. Furthermore, as shown in Fig 5, the scaling exponent is not obviously correlated with the warming rate in each month. This proves that the scaling exponent is an independent variable that represents another characteristic of the temperature time series and can play a unique role in the assessment of the climate system state and its variations.

### The long-range correlation of the annual mean temperature time series

**Fig 6:**
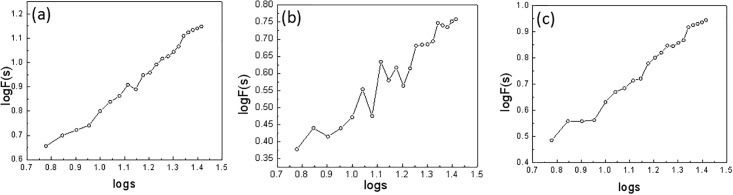
The long-range correlation of the annual mean temperatures from 1909–2014 for the stations at (a) Harbin; (b) Changchun; (c) Shenyang.

We further investigated the long-range correlation of the temperature time series at the annual time scale in the Northeast region (shown in Fig 6). From the figure, we can conclude that the centennial annual mean temperature time also shows a good scale distribution law with the calculated values of the scaling exponent *α*: 0.63, 0.75, and 0.82 for Harbin, Changchun, and Shenyang, respectively. Therefore, there is a good long-range correlation for the annual mean temperature time series with a certain predictability. At the same time, compared to the above part, the scaling exponent is smaller for the annual mean temperature time series than for the monthly mean temperature time series, with the same order of values for the three stations. This means that predicting the annual mean temperature is more difficult than predicting the monthly mean temperature to a certain degree and may be due to rather large interannual variations of the regional temperature.

### The long-range correlation of the monthly mean temperature during different time periods

From the previous analyses, the monthly mean temperature time series during the past 106 years in the Northeast region shows obvious long-range correlation characteristics, with less correlation with the warming rate. To answer the question whether the warming at the centennial scale changes the overall state of the Northeast region climate system, we divided the 106 years into two time periods of 1909–1961 and 1962–2014. We investigated the long-range correlation of the monthly mean temperatures at the three stations during these two time periods. The results are presented in Table 1. Regarding the Harbin station, the scaling exponent of the monthly mean temperature time series is 0.51 during 1909–1961 so that there is a weak long-range correlation during that time period with a random time series feature. However, its value is 0.71 during 1962–2014, which illustrates an obvious long-range correlation of the temperature time series during this time period and a stronger predictability of the monthly mean temperature. Regarding the Changchun station, the scaling exponent is 0.74 and 0.76 for the two time periods, respectively. The small difference proves that the predictability of the monthly mean temperature time series did not change much during the past one hundred years. The situation at the Shenyang station is similar to that of the Changchun station with the scaling exponent values of 0.80 and 0.85 for the two time periods, respectively, with a good long-range correlation.

**Table 1:** The scaling exponent of the monthly mean temperatures at different time periods from 1909–2014 for the three stations.

	Harbin	Changchun	Shenyang
*α* (1909–1961)	0.51±0.003	0.74±0.005	0.80±0.001
*α* (1962–2014)	0.71±0.006	0.76±0.004	0.85±0.003

The above results indicate that the warming affects different regions in different ways and may not change the predictability of the regional atmosphere. This situation may be due to how much the regional climate responds to global warming and needs further investigation.

## Conclusion and discussion

This study analyzed the centennial monthly mean temperature time series represented by the three stations at Harbin, Changchun, and Shenyang in the Northeast region of China. The research finds that there is long-range correlation in the monthly mean temperatures at the three stations studied, indicating that the temperature in the Northeast region has long-term memory and good predictability. In addition, the scaling exponent of the monthly mean temperature time series shows a weak predictability for March and April in the spring, but a better predictability for other months in the Northeast region. Furthermore, the research results also indicate long-range correlation features also present in the annual mean temperature time series with a very similar scale, as shown in the monthly mean temperatures. Finally, to investigate how warming affects the regional climate during different time periods, the centennial-scale temperature time series was divided into two periods: 1909–1960 and 1961–2014. We find that the long-range correlation at the Harbin station varies much during these two time periods but changes little at the other two stations. Therefore, warming affects the predictability of the regional climate system to a different degree at different time periods.

This paper initially analyzes the long-range correlation and evolution law of the centennial-scale monthly mean temperatures and provides a quantitative reference point for the assessment of regional climate predictability and future climate models. However, it is still necessary to further investigate the external forcing factors influencing the evolution of the regional long-range correlation and to improve regional temperature prediction based on this research work.

## Supporting information

S1 Data The data of three stations. (ZIP)
